# 3-(3-Chloro­anilino)-1-(3,5-dimethyl-1*H*-pyrazol-1-yl)propan-1-one

**DOI:** 10.1107/S1600536809016250

**Published:** 2009-05-07

**Authors:** Aamer Saeed, Shahid Hussain, Michael Bolte

**Affiliations:** a Department of Chemistry, Quaid-i-Azam University, Islamabad, Pakistan; b Institut für Anorganische Chemie, J. W. Goethe-Universität Frankfurt, Max-von-Laue-Strasse 7, 60438 Frankfurt/Main, Germany

## Abstract

In the mol­ecule of the title compound, C_14_H_16_ClN_3_O, the benzene and pyrazole rings are oriented at a dihedral angle of 3.50 (3)°. In the crystal structure, inter­molecular N—H⋯O hydrogen bonds link the mol­ecules into chains. A π–π contact between the benzene and pyrazole rings [centroid–centroid distance = 3.820 (3) Å] may further stabilize the structure.

## Related literature

For general background to 1,3,5-tris­ubstituted pyrazoles, see: Elguero & Goya (2002 ▶). The pyrazole chemotype is the structural motif of several highly potent inhibitors against coagulation factor Xa, see: Penning & Talley (1997 ▶); Eriksson & Quinlan (2006 ▶); Escolar *et al.* (2006 ▶). Pyrazole 3-carboxyl­ates have been identified as selective antagonist subtype 1PGE2 receptors (Akarca, 2005 ▶) and pyrazole-based mat­erials have been used as co-polymers for electroluminescent applications (Mella & Fagnoni, 1997 ▶). For the synthesis, see: Saeed & Mumtaz (2008 ▶). For bond-length data, see: Allen *et al.* (1987 ▶).
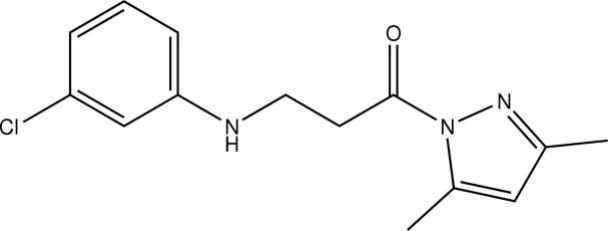


## Experimental

### 

#### Crystal data

C_14_H_16_ClN_3_O
*M* *_r_* = 277.75
Monoclinic, 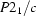

*a* = 14.5389 (8) Å
*b* = 7.8731 (6) Å
*c* = 12.1411 (7) Å
β = 102.566 (5)°
*V* = 1356.46 (15) Å^3^
*Z* = 4
Mo *K*α radiation
μ = 0.28 mm^−1^
*T* = 173 K
0.35 × 0.33 × 0.33 mm

#### Data collection

Stoe IPDS II two-circle diffractometer
Absorption correction: multi-scan (*MULABS*; Blessing, 1995 ▶) *T*_min_ = 0.909, *T*_max_ = 0.914
8907 measured reflections
2528 independent reflections
2192 reflections with *I* > 2σ(*I*)
*R*_int_ = 0.034

#### Refinement

*R*[*F* ^2^ > 2σ(*F* ^2^)] = 0.031
*wR*(*F* ^2^) = 0.086
*S* = 1.06
2528 reflections
179 parameters
H atoms treated by a mixture of independent and constrained refinement
Δρ_max_ = 0.22 e Å^−3^
Δρ_min_ = −0.23 e Å^−3^

### 

Data collection: *X-AREA* (Stoe & Cie, 2001 ▶); cell refinement: *X-RED* (Stoe & Cie, 2001 ▶); data reduction: *X-RED*; program(s) used to solve structure: *SHELXS97* (Sheldrick, 2008 ▶); program(s) used to refine structure: *SHELXL97* (Sheldrick, 2008 ▶); molecular graphics: *PLATON* (Spek, 2009 ▶); software used to prepare material for publication: *SHELXL97*.

## Supplementary Material

Crystal structure: contains datablocks I, global. DOI: 10.1107/S1600536809016250/hk2680sup1.cif

Structure factors: contains datablocks I. DOI: 10.1107/S1600536809016250/hk2680Isup2.hkl

Additional supplementary materials:  crystallographic information; 3D view; checkCIF report

## Figures and Tables

**Table 1:** Hydrogen-bond geometry (Å, °)

*D*—H⋯*A*	*D*—H	H⋯*A*	*D*⋯*A*	*D*—H⋯*A*
N1—H1⋯O1^i^	0.828 (18)	2.293 (19)	3.1101 (15)	169.1 (16)

Symmetry code: (i) 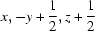.

**Fig. 1.**
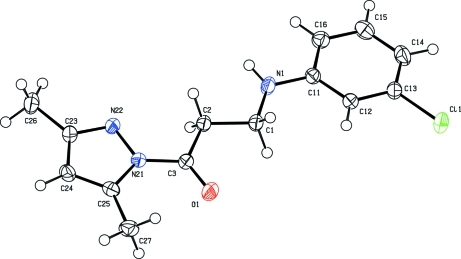
The molecular structure of the title molecule, with the atom-numbering scheme. Displacement ellipsoids are drawn at the 50% probability level.

**Fig. 2.**
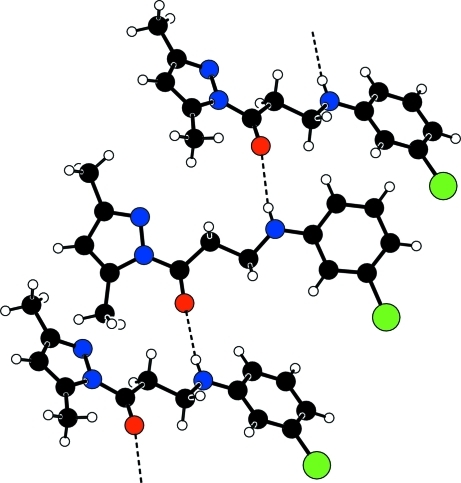
A partial packing diagram of the title compound. Hydrogen bonds are shown as dashed lines.

## supplementary crystallographic information

### Comment

1,3,5-Trisubstituted pyrazoles are synthetic targets of paramount significance in the pharmacological industry, in view of the fact that such a heterocyclic moiety represents the core structure of numerous drugs including the widely prescribed Celebrex and Viagra (Elguero & Goya, 2002). Pyrazole chemotype is structural motif of several highly potent inhibitors against coagulation factor Xa (Penning & Talley, 1997) among them Rivaroxaban (Eriksson & Quinlan, 2006) and Apixaban (Escolar *et al.*, 2006) were selected for clinical development for the prevention and treatment of thrombotic diseases. Pyrazole 3-carboxylates were also identified as selective antagonist subtype 1PGE2 receptors (Akarca, 2005). The pyrazole-based materials have been used as co-polymers for electroluminescent applications (Mella & Fagnoni, 1997). We report herein the crystal structure of the title  compound.

In the molecule of the title compound (Fig 1), the bond lengths (Allen *et al.*,  1987) and angles are within normal ranges. Rings A (C11-C16) and B (N21/N22/C23-C25) are, of course, planar, and they are oriented at a dihedral angle of A/B = 3.50 (3)°.

In the crystal structure, intermolecular N-H···O hydrogen bonds (Table 1) link the molecules into chains (Fig. 2), in which they may be effective in the stabilization of the structure. The π–π contact between the phenyl ring and the pyrazole ring, Cg1—Cg2^i^ [symmetry code: (i) 1 - x, 1 - y, -z, where Cg1 and Cg2 are centroids of the rings A (C11-C16) and B (N21/N22/C23-C25), respectively] may further stabilize the structure, with centroid-centroid distance of 3.820 (3) Å.

### Experimental

The title compound was prepared by cyclocondensation of pentane-2,4-dione with corresponding 3-(3-Chlorophenylamino) propionohydrazide according to a method reported earlier (Saeed & Mumtaz, 2008). Recrystallization from methanol afforded the title compound (yield; 81%). Anal. calcd. for C_14_H_16_ClN_3_O: C, 60.54; H, 5.81; N, 15.13%; found: C, 60.51; H, 5.83; N, 15.07%.

### Refinement

H atom of NH group was located in difference Fourier map and refined isotropically. The remaining H atoms were positioned geometrically with C-H = 0.95, 0.99 and 0.98 Å, for aromatic, methylene and methyl H atoms, respectively, and constrained to ride on their parent atoms, with U_iso_(H) = xU_eq_(C), where x = 1.5 for methyl H and x = 1.2 for all other H atoms.

### Crystal data

**Table:**

C_14_H_16_ClN_3_O	*F*(000) = 584
*M**_r_* = 277.75	*D*_x_ = 1.360 Mg m^−^^3^
Monoclinic, *P*2_1_/*c*	Mo *K*α radiation, λ = 0.71073 Å
Hall symbol: -P 2ybc	Cell parameters from 8296 reflections
*a* = 14.5389 (8) Å	θ = 3.5–25.9°
*b* = 7.8731 (6) Å	µ = 0.28 mm^−^^1^
*c* = 12.1411 (7) Å	*T* = 173 K
β = 102.566 (5)°	Block, orange
*V* = 1356.46 (15) Å^3^	0.35 × 0.33 × 0.33 mm
*Z* = 4	

### Data collection

**Table:**

Stoe IPDS II two-circle diffractometer	2528 independent reflections
Radiation source: fine-focus sealed tube	2192 reflections with *I* > 2σ(*I*)
graphite	*R*_int_ = 0.034
ω scans	θ_max_ = 25.6°, θ_min_ = 3.4°
Absorption correction: multi-scan (*MULABS*; Blessing, 1995)	*h* = −17→16
*T*_min_ = 0.909, *T*_max_ = 0.914	*k* = −9→8
8907 measured reflections	*l* = −14→14

### Refinement

**Table:**

Refinement on *F*^2^	Secondary atom site location: difference Fourier map
Least-squares matrix: full	Hydrogen site location: inferred from neighbouring sites
*R*[*F*^2^ > 2σ(*F*^2^)] = 0.031	H atoms treated by a mixture of independent and constrained refinement
*wR*(*F*^2^) = 0.086	*w* = 1/[σ^2^(*F*_o_^2^) + (0.0525*P*)^2^ + 0.155*P*] where *P* = (*F*_o_^2^ + 2*F*_c_^2^)/3
*S* = 1.06	(Δ/σ)_max_ < 0.001
2528 reflections	Δρ_max_ = 0.22 e Å^−^^3^
179 parameters	Δρ_min_ = −0.23 e Å^−^^3^
0 restraints	Extinction correction: *SHELXL97* (Sheldrick, 2008), Fc^*^=kFc[1+0.001xFc^2^λ^3^/sin(2θ)]^-1/4^
Primary atom site location: structure-invariant direct methods	Extinction coefficient: 0.0118 (15)

### Special details

**Table:**

Geometry. All e.s.d.'s (except the e.s.d. in the dihedral angle between two l.s. planes) are estimated using the full covariance matrix. The cell e.s.d.'s are taken into account individually in the estimation of e.s.d.'s in distances, angles and torsion angles; correlations between e.s.d.'s in cell parameters are only used when they are defined by crystal symmetry. An approximate (isotropic) treatment of cell e.s.d.'s is used for estimating e.s.d.'s involving l.s. planes.
Refinement. Refinement of *F*^2^ against ALL reflections. The weighted *R*-factor *wR* and goodness of fit *S* are based on *F*^2^, conventional *R*-factors *R* are based on *F*, with *F* set to zero for negative *F*^2^. The threshold expression of *F*^2^ > σ(*F*^2^) is used only for calculating *R*-factors(gt) *etc*. and is not relevant to the choice of reflections for refinement. *R*-factors based on *F*^2^ are statistically about twice as large as those based on *F*, and *R*- factors based on ALL data will be even larger.

### Fractional atomic coordinates and isotropic or equivalent isotropic displacement parameters (Å^2^)

**Table:**

	*x*	*y*	*z*	*U*_iso_*/*U*_eq_	
Cl1	0.09245 (2)	0.19275 (5)	0.40578 (3)	0.03790 (14)	
O1	0.56854 (7)	0.37061 (15)	0.32982 (8)	0.0338 (3)	
N1	0.44533 (8)	0.16939 (17)	0.58746 (10)	0.0303 (3)	
H1	0.4841 (13)	0.165 (2)	0.6485 (15)	0.035 (4)*	
C1	0.47221 (8)	0.24939 (17)	0.49191 (10)	0.0230 (3)	
H1A	0.4458	0.3656	0.4810	0.028*	
H1B	0.4478	0.1831	0.4224	0.028*	
C2	0.57897 (9)	0.25608 (17)	0.51596 (10)	0.0224 (3)	
H2A	0.6019	0.3236	0.5853	0.027*	
H2B	0.6041	0.1394	0.5304	0.027*	
C3	0.61688 (9)	0.33260 (16)	0.42090 (10)	0.0218 (3)	
C11	0.35323 (9)	0.13937 (17)	0.59318 (10)	0.0224 (3)	
C12	0.27731 (9)	0.18284 (17)	0.50608 (10)	0.0232 (3)	
H12	0.2873	0.2407	0.4410	0.028*	
C13	0.18671 (9)	0.13994 (18)	0.51620 (11)	0.0261 (3)	
C14	0.16813 (10)	0.0571 (2)	0.60922 (12)	0.0319 (3)	
H14	0.1056	0.0281	0.6135	0.038*	
C15	0.24443 (10)	0.01754 (19)	0.69646 (12)	0.0321 (3)	
H15	0.2337	−0.0378	0.7620	0.039*	
C16	0.33520 (10)	0.05718 (18)	0.68943 (11)	0.0270 (3)	
H16	0.3862	0.0289	0.7500	0.032*	
N21	0.71440 (8)	0.35624 (14)	0.44523 (8)	0.0211 (2)	
N22	0.76632 (8)	0.30431 (14)	0.54929 (9)	0.0234 (2)	
C23	0.85436 (9)	0.33838 (17)	0.54618 (11)	0.0252 (3)	
C24	0.86089 (9)	0.41270 (18)	0.44162 (11)	0.0261 (3)	
H24	0.9170	0.4479	0.4201	0.031*	
C25	0.77205 (9)	0.42387 (16)	0.37845 (10)	0.0228 (3)	
C26	0.93179 (10)	0.3031 (2)	0.64623 (13)	0.0374 (4)	
H26A	0.9551	0.4104	0.6828	0.056*	
H26B	0.9833	0.2441	0.6219	0.056*	
H26C	0.9078	0.2315	0.6998	0.056*	
C27	0.73737 (10)	0.49322 (19)	0.26314 (11)	0.0310 (3)	
H27A	0.7888	0.5528	0.2390	0.046*	
H27B	0.6855	0.5726	0.2636	0.046*	
H27C	0.7149	0.3999	0.2107	0.046*	

### Atomic displacement parameters (Å^2^)

**Table:**

	*U*^11^	*U*^22^	*U*^33^	*U*^12^	*U*^13^	*U*^23^
Cl1	0.01956 (19)	0.0532 (3)	0.0377 (2)	0.00375 (15)	−0.00099 (14)	−0.00656 (16)
O1	0.0241 (5)	0.0519 (7)	0.0233 (5)	−0.0005 (5)	0.0006 (4)	0.0057 (4)
N1	0.0177 (6)	0.0487 (8)	0.0228 (6)	−0.0042 (5)	0.0009 (5)	0.0071 (5)
C1	0.0182 (6)	0.0276 (7)	0.0228 (6)	−0.0011 (5)	0.0033 (5)	0.0014 (5)
C2	0.0181 (6)	0.0255 (7)	0.0227 (6)	−0.0010 (5)	0.0029 (5)	0.0007 (5)
C3	0.0197 (6)	0.0242 (7)	0.0209 (6)	0.0003 (5)	0.0030 (5)	−0.0023 (5)
C11	0.0203 (6)	0.0229 (6)	0.0243 (6)	−0.0023 (5)	0.0056 (5)	−0.0041 (5)
C12	0.0211 (6)	0.0259 (7)	0.0229 (6)	−0.0003 (5)	0.0055 (5)	−0.0026 (5)
C13	0.0194 (6)	0.0290 (7)	0.0292 (6)	0.0005 (5)	0.0038 (5)	−0.0078 (5)
C14	0.0234 (7)	0.0352 (8)	0.0401 (8)	−0.0051 (6)	0.0135 (6)	−0.0043 (6)
C15	0.0331 (8)	0.0337 (8)	0.0328 (7)	−0.0028 (6)	0.0145 (6)	0.0030 (6)
C16	0.0274 (7)	0.0286 (7)	0.0250 (6)	−0.0010 (6)	0.0058 (5)	0.0015 (5)
N21	0.0197 (5)	0.0255 (6)	0.0183 (5)	−0.0011 (4)	0.0044 (4)	0.0000 (4)
N22	0.0195 (5)	0.0299 (6)	0.0199 (5)	0.0003 (5)	0.0021 (4)	0.0019 (4)
C23	0.0193 (6)	0.0296 (7)	0.0263 (6)	0.0005 (5)	0.0042 (5)	−0.0025 (5)
C24	0.0222 (6)	0.0303 (7)	0.0279 (7)	−0.0047 (6)	0.0102 (5)	−0.0028 (5)
C25	0.0261 (6)	0.0215 (6)	0.0231 (6)	−0.0031 (5)	0.0104 (5)	−0.0032 (5)
C26	0.0199 (7)	0.0555 (10)	0.0345 (8)	0.0007 (7)	0.0012 (6)	0.0059 (7)
C27	0.0347 (7)	0.0370 (8)	0.0226 (6)	−0.0034 (6)	0.0090 (6)	0.0028 (6)

### Geometric parameters (Å, °)

**Table:**

Cl1—C13	1.7461 (14)	C14—H14	0.9500
O1—C3	1.2116 (16)	C15—C16	1.3767 (19)
N1—C11	1.3762 (17)	C15—H15	0.9500
N1—C1	1.4466 (16)	C16—H16	0.9500
N1—H1	0.828 (18)	N21—N22	1.3850 (15)
C1—C2	1.5165 (17)	N21—C25	1.3925 (16)
C1—H1A	0.9900	N22—C23	1.3164 (17)
C1—H1B	0.9900	C23—C24	1.4197 (18)
C2—C3	1.5085 (17)	C23—C26	1.4916 (19)
C2—H2A	0.9900	C24—C25	1.3540 (19)
C2—H2B	0.9900	C24—H24	0.9500
C3—N21	1.3964 (17)	C25—C27	1.4850 (18)
C11—C12	1.3950 (18)	C26—H26A	0.9800
C11—C16	1.4093 (18)	C26—H26B	0.9800
C12—C13	1.3909 (19)	C26—H26C	0.9800
C12—H12	0.9500	C27—H27A	0.9800
C13—C14	1.381 (2)	C27—H27B	0.9800
C14—C15	1.392 (2)	C27—H27C	0.9800
			
C11—N1—C1	123.43 (12)	C16—C15—H15	119.4
C11—N1—H1	115.4 (12)	C14—C15—H15	119.4
C1—N1—H1	119.0 (12)	C15—C16—C11	120.61 (13)
N1—C1—C2	107.73 (10)	C15—C16—H16	119.7
N1—C1—H1A	110.2	C11—C16—H16	119.7
C2—C1—H1A	110.2	N22—N21—C25	111.49 (10)
N1—C1—H1B	110.2	N22—N21—C3	118.72 (10)
C2—C1—H1B	110.2	C25—N21—C3	129.78 (11)
H1A—C1—H1B	108.5	C23—N22—N21	104.72 (10)
C3—C2—C1	113.30 (10)	N22—C23—C24	111.36 (11)
C3—C2—H2A	108.9	N22—C23—C26	120.32 (12)
C1—C2—H2A	108.9	C24—C23—C26	128.29 (12)
C3—C2—H2B	108.9	C25—C24—C23	106.98 (11)
C1—C2—H2B	108.9	C25—C24—H24	126.5
H2A—C2—H2B	107.7	C23—C24—H24	126.5
O1—C3—N21	121.39 (11)	C24—C25—N21	105.44 (11)
O1—C3—C2	124.12 (12)	C24—C25—C27	130.10 (12)
N21—C3—C2	114.49 (10)	N21—C25—C27	124.45 (12)
N1—C11—C12	122.53 (12)	C23—C26—H26A	109.5
N1—C11—C16	118.64 (12)	C23—C26—H26B	109.5
C12—C11—C16	118.81 (12)	H26A—C26—H26B	109.5
C13—C12—C11	118.80 (12)	C23—C26—H26C	109.5
C13—C12—H12	120.6	H26A—C26—H26C	109.5
C11—C12—H12	120.6	H26B—C26—H26C	109.5
C14—C13—C12	122.99 (13)	C25—C27—H27A	109.5
C14—C13—Cl1	118.68 (11)	C25—C27—H27B	109.5
C12—C13—Cl1	118.33 (11)	H27A—C27—H27B	109.5
C13—C14—C15	117.55 (13)	C25—C27—H27C	109.5
C13—C14—H14	121.2	H27A—C27—H27C	109.5
C15—C14—H14	121.2	H27B—C27—H27C	109.5
C16—C15—C14	121.21 (13)		
			
C11—N1—C1—C2	−178.65 (12)	O1—C3—N21—N22	−177.55 (12)
N1—C1—C2—C3	178.34 (11)	C2—C3—N21—N22	1.99 (16)
C1—C2—C3—O1	−6.68 (19)	O1—C3—N21—C25	1.0 (2)
C1—C2—C3—N21	173.80 (11)	C2—C3—N21—C25	−179.47 (12)
C1—N1—C11—C12	0.5 (2)	C25—N21—N22—C23	−0.40 (14)
C1—N1—C11—C16	178.65 (13)	C3—N21—N22—C23	178.39 (11)
N1—C11—C12—C13	176.66 (12)	N21—N22—C23—C24	0.26 (15)
C16—C11—C12—C13	−1.49 (19)	N21—N22—C23—C26	178.52 (12)
C11—C12—C13—C14	0.5 (2)	N22—C23—C24—C25	−0.02 (16)
C11—C12—C13—Cl1	−179.22 (10)	C26—C23—C24—C25	−178.12 (14)
C12—C13—C14—C15	0.7 (2)	C23—C24—C25—N21	−0.22 (14)
Cl1—C13—C14—C15	−179.52 (11)	C23—C24—C25—C27	179.06 (13)
C13—C14—C15—C16	−1.0 (2)	N22—N21—C25—C24	0.39 (14)
C14—C15—C16—C11	0.0 (2)	C3—N21—C25—C24	−178.23 (12)
N1—C11—C16—C15	−176.98 (13)	N22—N21—C25—C27	−178.94 (12)
C12—C11—C16—C15	1.2 (2)	C3—N21—C25—C27	2.4 (2)

### Hydrogen-bond geometry (Å, °)

**Table:**

*D*—H···*A*	*D*—H	H···*A*	*D*···*A*	*D*—H···*A*
N1—H1···O1^i^	0.828 (18)	2.293 (19)	3.1101 (15)	169.1 (16)

Symmetry codes: (i) *x*, −*y*+1/2, *z*+1/2.
